# Combined Predictive Value of GLIM-Defined Malnutrition and Preoperative Adipose Tissue ^18^F-FDG Uptake for Recurrence-Free Survival After Radical Gastrectomy in Patients with Gastric Cancer

**DOI:** 10.3390/curroncol32060363

**Published:** 2025-06-19

**Authors:** Xuan Zhou, Kailai Yin, Huanhuan Hong, Heqing Yi, Linfa Li

**Affiliations:** 1 Postgraduate Training Base Alliance of Wenzhou Medical University (Zhejiang Cancer Hospital), Hangzhou 310022, China; zhouxuan2025@sina.com (X.Z.); yinkailai1116@163.com (K.Y.); hihuan728@163.com (H.H.); 2 Department of Nuclear Medicine, Zhejiang Cancer Hospital, Hangzhou 310022, China; yiheqing1980@163.com

**Keywords:** gastric cancer, GLIM criteria, malnutrition, ^18^F-FDG PET/CT, adipose tissue, prognosis

## Abstract

Background: The Global Leadership Initiative on Malnutrition (GLIM) criteria provide a standardized approach for assessing the nutritional status of patients and demonstrate strong predictive value for the prognosis of patients with gastric cancer. However, these criteria do not incorporate indicators of adipose tissue metabolic activity, which may reflect pro-tumor microenvironmental factors. This study investigated the combined predictive value of malnutrition, defined by the GLIM criteria, and preoperative adipose tissue ^18^F-fluorodeoxyglucose (^18^F-FDG) uptake for recurrence-free survival (RFS) in patients with gastric cancer following radical surgery. Methods: A total of 105 patients were retrospectively enrolled and classified into malnourished and non-malnourished groups based on the GLIM criteria. Preoperative ^18^F-FDG positron emission tomography/computed tomography (^18^F-FDG PET/CT) was used to measure the mean standardized uptake value (SUVmean) of visceral adipose tissue (VAT) and subcutaneous adipose tissue (SAT). The predictive values of these indicators for RFS in patients with gastric cancer were assessed. Results: Multivariate survival analysis was used to identify GLIM-defined malnutrition (*p* = 0.020) and increased preoperative VAT SUVmean (*p* = 0.042) as independent risk factors for RFS. The combined analysis revealed that patients with both malnutrition and a high preoperative VAT SUVmean had the poorest RFS (HR = 18.41, *p* < 0.001). The predictive model integrating GLIM criteria and VAT SUVmean outperformed the GLIM criteria alone. Conclusions: This study demonstrated that combining malnutrition defined by the GLIM criteria with preoperative visceral adipose tissue ^18^F-FDG uptake optimizes recurrence risk stratification and exhibits superior prognostic predictive efficacy compared to using the GLIM criteria alone. This approach provides new insights into individualized prognostic assessment and intervention strategies.

## Simple Summary

Predicting the risk of postoperative recurrence in patients with gastric cancer is highly valuable for facilitating diagnostic and treatment decisions. The Global Leadership Initiative on Malnutrition criteria provide a standardized approach for assessing the nutritional status of patients and demonstrate strong predictive value for the prognosis of patients with gastric cancer. However, these criteria do not incorporate indicators of adipose tissue metabolic activity, which may reflect pro-tumor microenvironmental factors. Recent studies have increasingly demonstrated associations between ^18^F-fluorodeoxyglucose uptake in the adipose tissue and the clinical staging or prognosis of various malignancies. This study demonstrated that combining malnutrition defined by the GLIM criteria with preoperative visceral adipose tissue ^18^F-fluorodeoxyglucose uptake optimizes the recurrence risk stratification and exhibits superior prognostic predictive efficacy compared to using the GLIM criteria alone. This approach provides new insights into individualized prognostic assessment and intervention strategies.

## 1. Introduction

Gastric cancer is the fifth most common malignancy and the third leading cause of cancer-related mortality [1,2]. The occurrence of gastric cancer is associated with multiple factors, among which *Helicobacter pylori* infection is recognized as the most significant preventable risk factor [1]. When considering tumor invasion depth, gastric cancer can be classified into early gastric cancer (where cancer tissue is limited to the mucosa or submucosa, regardless of the presence or absence of lymph node metastasis) and advanced gastric cancer (where cancer tissue infiltrates beyond the submucosa, penetrates the muscularis propria, or extends through the muscularis propria to the serosa) [1]. For patients with non-metastatic gastric cancer, radical gastrectomy combined with adjuvant chemotherapy or chemoradiotherapy remains the standard treatment. However, postoperative local recurrence and distant metastasis are common [3]. Therefore, the accurate assessment of recurrence risk and prognosis (defined as the prediction of a disease’s future course and outcome) in patients with gastric cancer after surgery is important to guide the selection of an optimal treatment plan and improve the patient’s quality of life.

In recent years, multiple studies have demonstrated that the preoperative nutritional status of patients with gastric cancer has a significant prognostic value [4,5,6]. Although various nutritional risk screening tools have been developed internationally [7,8,9], globally accepted diagnostic criteria for malnutrition are lacking. The Global Leadership Initiative on Malnutrition (GLIM) consensus [10] proposed standardized global diagnostic criteria for malnutrition and clarified the phenotypic and etiological metrics used in diagnosing malnutrition. Studies using the GLIM criteria revealed that the incidence of preoperative malnutrition in patients with gastric cancer can reach up to 50%. Compared to patients with normal nutritional status, those with pre-existing malnutrition before treatment exhibit poorer prognoses [11,12].

Adipose tissue can be classified into subcutaneous adipose tissue (SAT) and visceral adipose tissue (VAT) based on its anatomical location. Adipocytes contribute to tumor cell invasion and metastasis by secreting adipokines, maintaining an inflammatory state and facilitating angiogenesis [13,14]. ^18^F-fluorodeoxyglucose positron emission tomography/computed tomography (^18^F-FDG PET/CT), a noninvasive functional imaging technique, has been widely used in tumor diagnosis, staging, and treatment efficacy evaluation. The ^18^F-FDG uptake in adipose tissue reflects the glucose metabolism in adipocytes and inflammatory cells infiltrating the tissue; moreover, it can reflect the characteristics of the adipose microenvironment promoting tumor growth and aggressiveness [15,16]. Recent studies have increasingly demonstrated associations between ^18^F-FDG uptake in the adipose tissue and the clinical staging or prognosis of various malignancies. However, these results are contradictory. The majority of studies indicate that high ^18^F-FDG uptake in adipose tissue is a risk factor for patient prognosis [15,16,17,18]; however, a few studies have arrived at contrary findings [19]. Systematic investigations of gastric cancer remain scarce. Notably, emerging evidence suggests a strong correlation between preoperative adipose tissue ^18^F-FDG uptake and nutritional status in patients with gastric cancer [20]; however, the current GLIM nutritional assessment criteria do not incorporate adipose tissue-related parameters. Consequently, integrating adipose tissue ^18^F-FDG uptake with the GLIM-based nutritional evaluation is expected to enhance the predictive performance of the GLIM criteria and is of significant value for patient prognostic stratification and the formulation of individualized treatment plans.

To date, no studies have explored the synergistic predictive value of combining adipose tissue ^18^F-FDG uptake with the GLIM criteria for recurrence-free survival (RFS) in patients with gastric cancer following curative resection. In addition, the predictive performance of adipose tissue ^18^F-FDG uptake levels for the prognosis of patients with gastric cancer has not been fully validated. Therefore, this study aimed to evaluate the correlation between the mean standardized uptake value (SUVmean) of VAT and SAT, preoperative malnutrition status based on the GLIM criteria, and RFS in patients with gastric cancer following radical gastrectomy. Furthermore, we investigated whether assessing adipose tissue metabolism using PET/CT could enhance the predictive performance of the GLIM criteria for RFS. These findings could provide novel insights for the establishment of a precise prognostic prediction system for gastric cancer.

## 2. Materials and Methods

### 2.1. Patient Selection

This study retrospectively analyzed patients with gastric cancer who underwent radical gastrectomy from September 2019 to September 2023. Patients were included in this study who (1) were treated with radical gastrectomy and postoperatively confirmed as having gastric cancer by histopathology, for whom the decision regarding adjuvant chemotherapy was based on their condition (including the 8th edition American Joint Committee on Cancer [AJCC] Tumor Node Metastasis [TNM] stage, postoperative pathological characteristics, immunohistochemistry results, and patient performance status), with their adjuvant chemotherapy regimens (the chemotherapy regimens primarily consisted of the following: (1) SOX chemotherapy consisting of oxaliplatin and S-1 and (2) XELOX chemotherapy consisting of oxaliplatin and capecitabine) following the National Comprehensive Cancer Network (NCCN) Guidelines and the Chinese Society of Clinical Oncology (CSCO) Guidelines; and (2) had complete preoperative PET/CT imaging data. Meanwhile, those who had (1) concurrent primary malignancies; (2) severe preoperative cardiovascular/cerebrovascular diseases, hepatic/renal insufficiency, hyperthyroidism, or diabetes mellitus; (3) received radiotherapy, chemotherapy, or other medical treatments prior to surgery; and (4) incomplete clinical data were excluded. In total, 105 patients who met the inclusion criteria were selected as the study cohort. This study was approved by the Ethics Committee of Zhejiang Cancer Hospital (approval number: IRB-2025-425).

### 2.2. ^18^F-FDG PET/CT Image Acquisition

This study strictly adhered to the current guidelines for ^18^F-FDG PET/CT examination [21]. The standardized protocol was implemented as follows: Patients fasted for 6–8 h prior to imaging, with blood glucose levels controlled to ≤8.3 mmol/L. An intravenous injection of ^18^F-FDG (3.7 MBq/kg) was administered, followed by a resting period of 60 ± 10 min in a light-restricted environment. Prior to PET/CT (GE Discovery PET/CT 710, Biograph Vision PET/CT) scanning, the patients were instructed to empty their bladders and consume 500 mL of water to adequately distend the gastric lumen. The imaging coverage extended from the cranial vertex to the upper femoral region. Dedicated acquisition protocols were implemented, with variable durations: 2–3 min per bed position for the trunk and 8–10 min per bed position for the head. Concurrent CT was performed using the following parameters: 120 kV, 100 mA, and a scan thickness of 3 mm. Attenuation correction was conducted based on the CT data, and images were reconstructed using an iterative algorithm to generate whole-body PET, CT, and PET/CT fusion images.

### 2.3. Image Analysis and Data Collection

**Figure 1:**
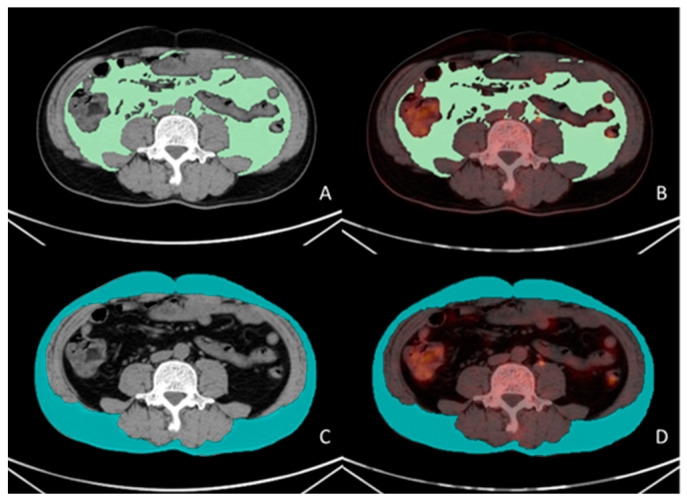
Measurement methodology for VAT and SAT SUVmean. A 56-year-old male patient with gastric cancer underwent preoperative ^18^F-FDG PET/CT examination. Visualized using LIFEx software (version 7.4.0). VAT (green color in (**A**)) and SAT (blue color in (**C**)) regions were delineated on three consecutive CT slices at the L4–L5 vertebral level based on the CT attenuation range (−50 to −150 Hounsfield units [HU]). The CT-defined VAT and SAT regions were subsequently co-registered with corresponding PET/CT fusion images (green color in (**B**), blue color in (**D**)), yielding a VAT SUVmean of 0.43 and an SAT SUVmean of 0.32.

**Figure 2:**
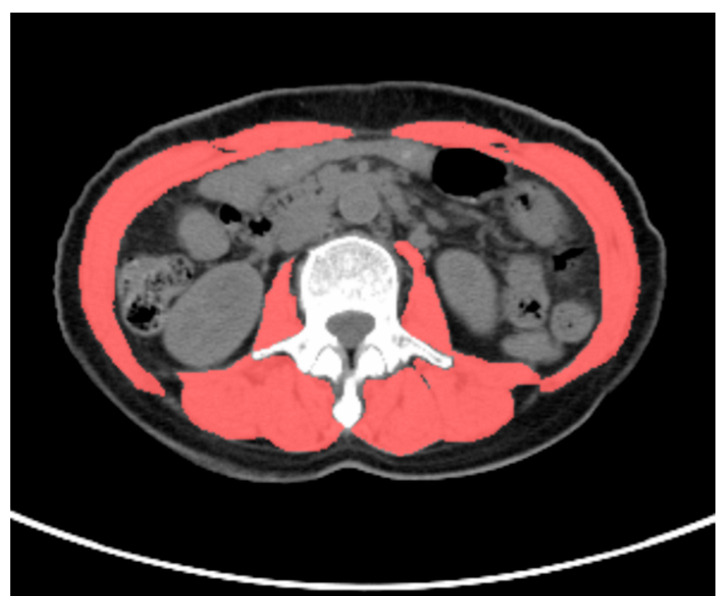
Methodology for measuring skeletal muscle cross-sectional area. A 58-year-old female patient with gastric cancer underwent preoperative ^18^F-FDG PET/CT examination. The total skeletal muscle cross-sectional area at the L3 vertebral level was delineated based on a CT attenuation range (−29 HU to 150 HU (scarlet color )) on CT images using LIFEx software version 7.4.0. The total skeletal muscle cross-sectional area at the L3 vertebral level in this patient was 91.2 cm^2^.

All PET/CT images were delineated by two nuclear medicine physicians, who were blinded to the clinical data. The VAT SUVmean, SAT SUVmean, and skeletal muscle cross-sectional area were obtained using the LIFEx software version 7.4.0. VAT and SAT regions at the L4–L5 vertebral level across three consecutive CT slices were delineated based on CT attenuation values, ranging from −50 Hounsfield units (HU) to −150 HU [13]. These CT-defined VAT and SAT regions were mapped onto the corresponding PET/CT fusion images to acquire the VAT SUVmean and SAT SUVmean, as shown in Figure 1. The total skeletal muscle cross-sectional area at the L3 level (including the psoas major, erector spinae, quadratus lumborum, transversus abdominis, internal oblique, external oblique, and rectus abdominis muscles) was delineated based on the CT attenuation range (−29 HU to 150 HU) on CT images, as shown in Figure 2. The skeletal muscle index (SMI) at L3 was calculated as follows: skeletal muscle cross-sectional area at L3 (cm^2^)/height squared (m^2^) [22].

Preoperative patient information, including demographic characteristics such as sex, age, and preoperative body mass index (BMI), was retrieved from hospital databases. Preoperative hematological parameters, including albumin, prealbumin, platelet count, neutrophil count, lymphocyte count, and C-reactive protein levels, were also collected. Disease staging was reassessed according to the 8th edition of the TNM classification system and pathological findings, and the number of intraoperatively examined lymph nodes was recorded. Additionally, the surgical procedure performed on each patient, whether adjuvant chemotherapy was administered postoperatively, and details of early postoperative complications (postoperative hemorrhage, anastomotic leakage, intestinal obstruction, and infection) were comprehensively documented. Postoperative follow-up was conducted for all patients through outpatient reviews or telephone interviews, with the follow-up cutoff date set in April 2025. The study endpoint was RFS, defined as the interval from postoperative day 1 after radical surgery until either the first documented tumor recurrence or the end of follow-up.

### 2.4. GLIM Criteria

The preoperative nutritional status of enrolled patients was assessed according to the GLIM criteria [10,23]. The diagnostic process comprised two sequential steps: initial nutritional risk screening using validated nutritional screening tools, followed by a comprehensive diagnostic evaluation integrating phenotypic and etiologic criteria for patients with nutritional risk. The phenotypic criteria included non-volitional weight loss (>5% within 6 months or >10% beyond 6 months), low BMI (<18.5 kg/m^2^ for individuals aged <70 years or <20 kg/m^2^ for those aged ≥70 years), and reduced skeletal muscle mass (skeletal muscle index <52.4 cm^2^/m^2^ for men and <38.5 cm^2^/m^2^ for women). The etiologic criteria included reduced food intake or assimilation (≤50% of energy requirement for >1 week, <75% for >2 weeks, or the presence of gastrointestinal disorders impairing absorption) and inflammatory conditions (acute injury/illness or chronic inflammation associated with malignancies). Patients meeting at least one phenotypic criterion and one etiologic criterion were classified into the malnutrition group, whereas those who did not meet these composite criteria were assigned to the non-malnutrition group. The above process for assessing malnutrition according to the GLIM criteria was performed by nuclear medicine physicians.

### 2.5. Statistical Analysis

**Figure 3:**
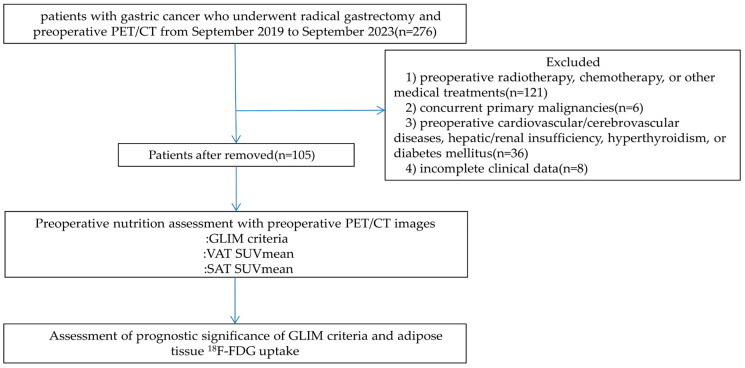
Schematic overview of the overall workflow in this study.

A schematic presentation of the workflow in the present study is depicted in Figure 3. Statistical analyses were performed using R 4.0.2 and SPSS 27.0. Continuous variables are expressed based on the Shapiro–Wilk test results. Normally distributed data were compared between the groups using Student’s *t*-test, whereas non-normally distributed data were analyzed using the Mann–Whitney U test. Categorical variables were compared using the Chi-square test or Fisher’s exact test, as appropriate. Spearman’s rank correlation test was used to assess intervariable correlations. Univariate Cox regression analysis was conducted to preliminarily screen for recurrence-free survival (RFS)-associated factors, followed by multivariate Cox proportional hazards models to identify independent prognostic factors. Survival curves were generated using the Kaplan–Meier method, with between-group survival differences evaluated using log-rank tests. A combined GLIM criteria–VAT SUVmean model was constructed for RFS prediction. Model performance comparisons among the GLIM criteria, VAT SUVmean, and the combined model were conducted using likelihood ratio tests (LRTs), the Akaike Information Criterion (AIC), and concordance indexes (C-indexes). Larger LRT χ^2^ values, smaller AIC values, and higher C-index values indicated superior model performance. All tests were two-tailed, and statistical significance was defined as a *p*-value of <0.05.

## 3. Results

### 3.1. Patient Characteristics

The baseline characteristics of the 105 enrolled patients are summarized in Table 1. VAT SUVmean and SAT SUVmean values from 105 patients with gastric cancer were sorted in ascending order, yielding median values of 0.41 and 0.33, respectively. Based on the median values, the patients were stratified into the high VAT SUVmean (≥0.41, n = 53, 50.5%), low VAT SUVmean (<0.41, n = 52, 49.5%), high SAT SUVmean (≥0.33, n = 53, 50.5%), and low SAT SUVmean (<0.33, n = 52, 49.5%) groups. Among these patients, 41 (39.0%) were diagnosed with malnutrition according to the GLIM criteria, whereas 64 (61.0%) were classified as not malnourished. Lymph node metastasis was detected in 66 patients (62.9%), of whom 53 (50.5%) had AJCC stage III disease. Fifty patients (47.6%) had poorly differentiated tumors. Total gastrectomy was performed in 47 (44.8%) patients. Postoperative adjuvant chemotherapy was administered to 70 (66.7%) patients.

The median postoperative follow-up duration was 23 months (range: 10–67 months), during which 34 patients experienced recurrence. Comparative analysis between the recurrence and non-recurrence groups revealed that the preoperative BMI was significantly lower in the recurrence group (*p* = 0.01). The recurrence group demonstrated a significantly higher preoperative malnutrition prevalence, VAT SUVmean, and SAT SUVmean compared to the non-recurrence group (*p* < 0.05). Moreover, the recurrence group had advanced tumor stages (*p* < 0.001), a high proportion of patients who received adjuvant chemotherapy (*p* = 0.018), and a high proportion of patients who underwent total gastrectomy (*p* = 0.045). With regard to preoperative hematological parameters, albumin and prealbumin levels were significantly higher in the non-recurrence group (*p* < 0.01), whereas neutrophil-to-lymphocyte ratio (NLR), platelet-to-lymphocyte ratio (PLR), and C-reactive protein (CRP) levels were significantly lower in the non-recurrence group (*p* < 0.01).

### 3.2. Correlation Analysis Between VAT SUVmean/SAT SUVmean and Clinical Data

**Figure 4:**
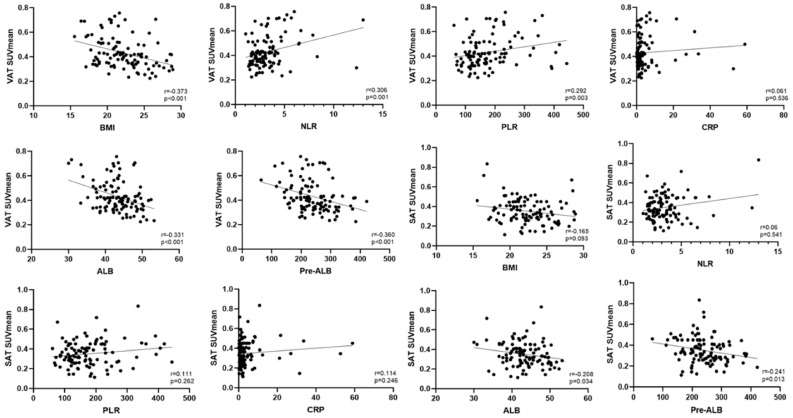
Scatter plots illustrating the correlations between VAT SUVmean and SAT SUVmean with clinical data.

The correlation analysis of the VAT SUVmean and SAT SUVmean with clinical data demonstrated that VAT SUVmean showed statistically significant negative correlations with BMI, and albumin and prealbumin levels (*p* < 0.001). The VAT SUVmean exhibited statistically significant positive correlations with the NLR and PLR (*p* < 0.05). SAT SUVmean only displayed a statistically significant negative correlation with albumin and prealbumin levels (*p* < 0.05) (Table 2 and Figure 4).

### 3.3. Survival Analysis for RFS

Univariate and multivariate Cox regression analyses were performed to evaluate the prognostic value of GLIM-defined malnutrition and adipose tissue ^18^F-FDG uptake for RFS. Univariate Cox regression analysis identified GLIM-defined malnutrition (*p* < 0.001), low BMI (*p* = 0.023), advanced tumor stage (*p* < 0.001), a VAT SUVmean of ≥0.41 (*p* < 0.001), an SAT SUVmean of ≥0.33 (*p* = 0.034), hypoalbuminemia (*p* = 0.001), low prealbumin levels (*p* < 0.001), elevated NLR levels (*p* = 0.016), elevated PLR levels (*p* = 0.006), adjuvant chemotherapy (*p* = 0.018), and total gastrectomy (*p* = 0.031) as RFS risk factors. Multivariate Cox regression analysis confirmed that GLIM-defined malnutrition (*p* = 0.020) and VAT SUVmean ≥0.41 (*p* = 0.042) were independent risk factors for RFS (Table 3).

**Figure 5:**
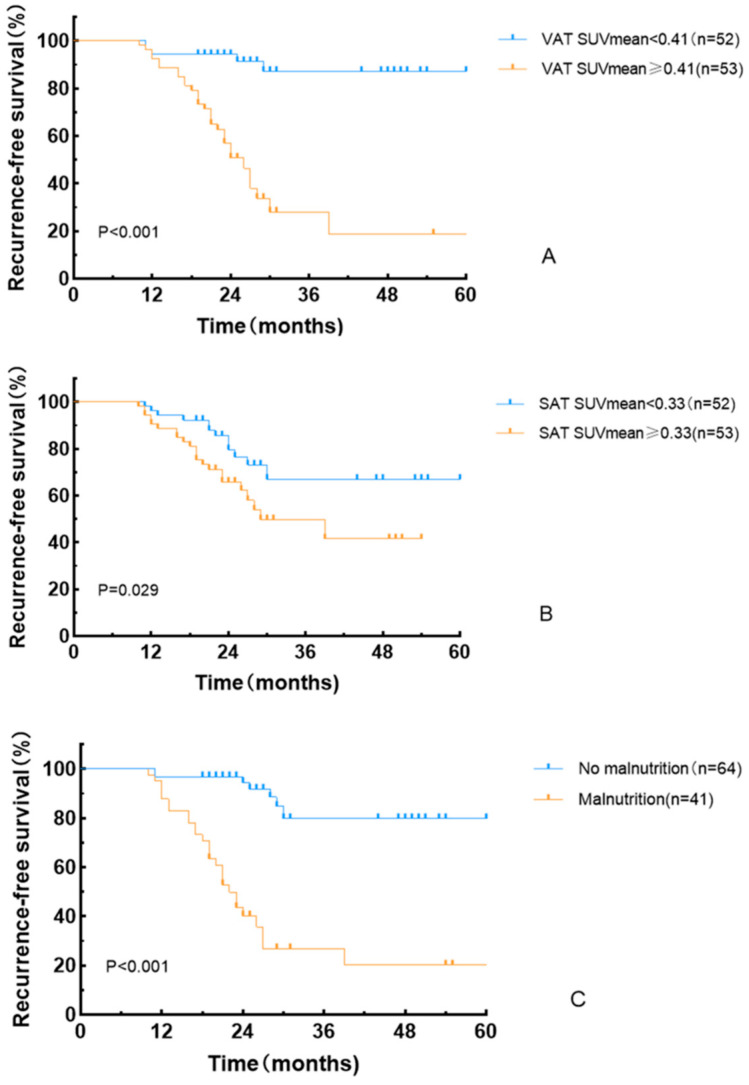
Recurrence-free survival curves based on VAT SUVmean (**A**), SAT SUVmean (**B**), and GLIM criteria (**C**).

Kaplan–Meier analysis demonstrated a significantly shorter RFS in the high VAT SUVmean group compared to the low VAT SUVmean group (log-rank χ^2^ = 28.457, *p* < 0.001), in the high SAT SUVmean group compared to the low SAT SUVmean group (log-rank χ^2^ = 4.766, *p* = 0.029), and in the malnutrition group compared to the non-malnutrition group (log-rank χ^2^ = 39.805, *p* < 0.001) (Figure 5).

### 3.4. Stratification of RFS by Combining the GLIM Criteria and the VAT SUVmean

**Figure 6:**
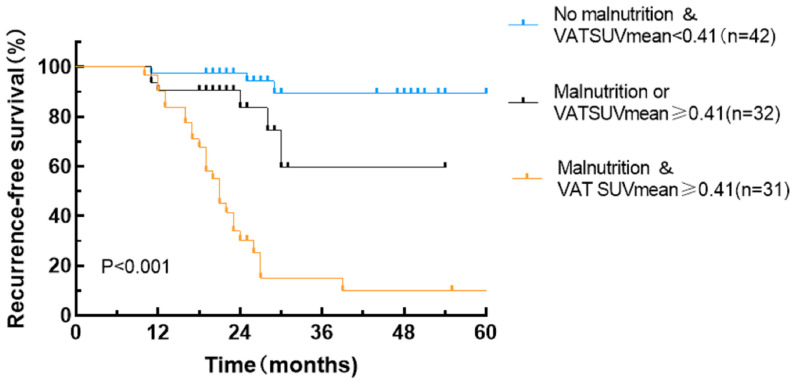
Recurrence-free survival curves stratified by combining the GLIM criteria and the VAT SUVmean.

By integrating the GLIM criteria with VAT SUVmean, patient prognoses were further stratified into three distinct RFS subgroups exhibiting significant differences in outcomes: patients with concurrent malnutrition and a VAT SUVmean of ≥0.41, patients with either malnutrition or a VAT SUVmean of ≥0.41, and patients without malnutrition and a VAT SUVmean of <0.41 (Table 4 and Figure 6). Specifically, the malnutrition with VAT SUVmean ≥0.41 group (*p* < 0.001) and the malnutrition or VAT SUVmean ≥0.41 group (*p* < 0.001) demonstrated significantly lower RFS rates compared to the group without malnutrition and with a VAT SUVmean of <0.41.

### 3.5. Predictive Value of the GLIM Criteria–VAT SUVmean Model in the Postoperative Prognosis of Patients with Gastric Cancer

For the prediction of RFS, the GLIM criteria–VAT SUVmean model demonstrated larger χ^2^ values, smaller AIC values, and higher C-indexes compared to the GLIM criteria or VAT SUVmean alone (all *p* < 0.001; Table 5). These results indicate that the combined model outperformed the GLIM criteria and VAT SUVmean alone in predicting RFS.

## 4. Discussion

This study evaluated the association between preoperative adipose tissue ^18^F-FDG uptake, preoperative malnutrition status assessed based on the GLIM criteria, and RFS in patients with gastric cancer after radical resection. This study is the first to report the synergistic predictive value of their combined application for postoperative RFS. GLIM criteria-diagnosed malnutrition, based on phenotypic and etiologic criteria, has been significantly correlated with the prognosis of various malignancies [24,25]. In this study, GLIM-defined malnutrition was identified as an independent predictor of RFS (*p* = 0.020) in patients with gastric cancer undergoing radical surgery, consistent with previous research findings. In this study, the univariate Cox regression analysis showed that adjuvant chemotherapy was associated with an increased risk of recurrence (which may be related to the tendency of advanced-stage patients to receive adjuvant chemotherapy; *p* = 0.018); however, its independent predictive value was limited (*p* = 0.170). However, this does not negate the predictive value of adjuvant chemotherapy for the prognosis of gastric cancer, but rather suggests, to some extent, that its efficacy may be significantly influenced by tumor burden and patient nutritional status. Malnourished patients often require chemotherapy dose reductions, delays, or discontinuation due to treatment-related toxicities (such as myelosuppression, increased risk of infection, and severe gastrointestinal reactions), which lead to reduced treatment intensity and, consequently, a poor prognosis [26,27]. This study did not present an in-depth analysis of the impact of the quality of chemotherapy completion (e.g., dose intensity and treatment course completion) on the prognosis of patients with gastric cancer. This may represent a key mechanism through which malnutrition affects outcomes and warrants further investigation in future studies. Notably, recent studies have indicated that the exclusion of adipose tissue-related parameters from the GLIM criteria may compromise its diagnostic and prognostic predictive performance [25,28,29]. Several studies on gastric cancer have shown that the incorporation of adipose tissue-related parameters significantly enhances the prognostic predictive efficacy of the GLIM criteria compared to its use alone [28,30].

For decades, adipose tissue has been regarded as a passive energy storage organ. However, current evidence has demonstrated that adipose tissue functions as a highly heterogeneous endocrine organ capable of secreting diverse bioactive substances [31,32]. Emerging evidence has revealed bidirectional interactions between adipocytes and tumor cells, where neoplastic cells induce morphological alterations in adjacent adipocytes, characterized by reduced cell volume, dispersed lipid droplets, and the acquisition of a fibroblast-like phenotype, ultimately leading to the formation of cancer-associated adipocytes (CAAs) [14,33,34]. These CAAs secrete multiple factors and establish an inflammatory microenvironment within the adipose tissue, thereby promoting tumor cell proliferation, invasion, and metastasis. Previous studies have revealed that ^18^F-FDG uptake in adipose tissue is significantly associated with tumor stage and patient prognosis; however, these findings are conflicting. Specifically, investigations in colorectal cancer cohorts and a separate study in patients with breast cancer documented markedly elevated VAT ^18^F-FDG uptake in advanced-stage patients, with high VAT SUVmean levels identified as an independent risk factor for survival, consistent with the results of our study [17,35]. However, a study in patients with pancreatic cancer demonstrated that those with lymph node or distant metastases had significantly lower ^18^F-FDG uptake in SAT than those without metastases, likely owing to a mechanism through which advanced tumor cells inhibit glucose metabolism in adipocytes to obtain more exogenous fatty acids for tumor growth [19]. The authors of these studies demonstrated that ^18^F-FDG uptake in the VAT is associated with macrophage-induced inflammatory responses within the adipose tissue. Given the crucial roles of both CAAs and macrophages within the tumor microenvironment, ^18^F-FDG uptake in VAT may also indirectly reflect the interactions between adipocytes and tumor cells.

This study demonstrated that a VAT SUVmean of ≥0.41 was an independent risk factor for RFS in patients with gastric cancer (*p* = 0.042), whereas an SAT SUVmean of ≥0.33 showed no independent prognostic significance (*p* = 0.434). Previous studies have consistently reported higher ^18^F-FDG uptake in VAT compared with SAT in both patients with malignant tumors and healthy individuals [17,35,36], which is consistent with the findings of our study. The metabolic hyperactivity of VAT relative to SAT may be attributed to two primary mechanisms. First, VAT exhibits the higher expression of lipolysis-promoting beta-adrenergic receptors and the lower expression of inhibitory alpha-adrenergic receptors, enhancing lipid breakdown [37]. Second, VAT demonstrates the greater infiltration of inflammatory cells (e.g., lymphocytes and macrophages) and increased secretion of pro-inflammatory cytokines such as interleukin-6 and tumor necrosis factor-alpha [38]. These findings suggest that VAT SUVmean may serve as a more clinically significant imaging biomarker for gastric cancer prognosis compared to SAT SUVmean.

Our study demonstrates that integrating malnutrition, defined based on the GLIM criteria, with visceral adipose tissue VAT ^18^F-FDG uptake enables the refined stratification of cancer recurrence risk in patients with gastric cancer. The preoperative assessment identified the subgroup with malnutrition and a VAT SUVmean of ≥0.41 as having the poorest prognosis. Meanwhile, the subgroup without malnutrition and a VAT SUVmean of <0.41 exhibited optimal outcomes. Furthermore, a novel GLIM-VAT SUVmean predictive model was established by combining these parameters. Through a comparative analysis using LRT, AIC, and C-index, the GLIM-VAT SUVmean model demonstrated superior predictive performance for RFS compared to the individual GLIM criteria or VAT SUVmean alone. These findings confirm that VAT ^18^F-FDG uptake enhances the prognostic capability of the GLIM criteria for RFS prediction. Our analysis revealed significant negative correlations between VAT SUVmean and nutritional markers (albumin and prealbumin), which may be associated with increased energy expenditure during malnutrition [20,39]. Simultaneously, VAT SUVmean positively correlated with inflammatory indexes (NLR and PLR). However, the existing literature displays conflicting findings regarding these correlations, with one study reporting that only PLR was significantly correlated with VAT SUVmean [15], while another study revealed no significant correlation between VAT SUVmean and either NLR or PLR [16]. Therefore, the precise relationship between the VAT SUVmean and systemic inflammatory markers requires further investigation. It is speculated that VAT SUVmean may serve as a significant indicator for assessing both nutritional and inflammatory status in patients with gastric cancer. Consequently, the preoperative metabolic assessment of VAT not only optimizes recurrence risk prediction but also provides imaging-based evidence to support the development of personalized nutritional interventions.

However, this study has some limitations. The retrospective design, small sample size, and single-center nature of this study necessitate further validation through large-scale multi-center studies. This study documented whether patients received postoperative adjuvant chemotherapy, but did not comprehensively evaluate the completion rates of chemotherapy or the relative dose intensity, both of which may be significantly influenced by nutritional status. Future studies should incorporate metrics for chemotherapy compliance to elucidate the mechanisms by which malnutrition affects prognosis more precisely. Additionally, histopathological and laboratory analyses should be conducted to elucidate the mechanisms underlying these findings. Currently, no standardized method exists for measuring the adipose tissue SUVmean. Future research should develop standardized measurement protocols that accurately capture the qualitative characteristics of adipose tissue. Notably, emerging artificial intelligence technologies, particularly the application of deep learning methods in medical image segmentation and quantitative analysis, present significant potential for automating adipose tissue identification, accurately delineating regions of interest, and reducing inter-observer variability. Artificial intelligence technology holds promise as a key tool for achieving a standardized, cross-centered, and reproducible assessment of the adipose tissue metabolic activity.

## 5. Conclusions

In conclusion, preoperative malnutrition, defined based on the GLIM criteria, and elevated preoperative VAT SUVmean were identified as independent risk factors for RFS in patients with gastric cancer undergoing radical surgery. The combined application of these parameters significantly improved recurrence risk stratification, with the integrated model demonstrating superior prognostic predictive efficacy compared to the individual GLIM criteria or VAT SUVmean alone. These findings provide novel insights for developing personalized prognostic evaluation frameworks and formulating precise nutritional intervention strategies in gastric cancer management.

## Figures and Tables

**Table 1:** Patient characteristics.

Characteristics	Total (n = 105)	Recurrence (n = 34)	No Recurrence (n = 71)	*p*-Value
Age (years)	63 (23–88)	63 (34–88)	64 (23–81)	0.696
BMI (kg/m^2^)	22.1 (15.55–28.89)	20.9 (15.55–28.54)	22.8 (17.12–28.89)	0.01 *
Sex				0.434
Men	82 (78.1%)	25 (73.5%)	57 (80.3%)	
Women	23 (21.9%)	9 (26.5%)	14 (19.7%)	
T stage				<0.001 *
T1–2 stage	37 (35.2%)	2 (6%)	35 (49.3%)	
T3–4 stage	68 (64.8%)	32 (94%)	36 (50.7%)	
N stage				<0.001 *
N0 stage	39 (37.1%)	5 (14.7%)	34 (47.9%)	
N1–3 stage	66 (62.9%)	29 (85.3%)	37 (52.1%)	
AJCC stage				<0.001 *
I-II	52 (49.5%)	8 (23.5%)	44 (62.0%)	
III	53 (50.5%)	26 (76.5%)	27 (38.0%)	
Adjuvant chemotherapy				0.018 *
Yes	70 (66.7%)	28 (82.4%)	42 (59.2%)	
No	35 (33.3%)	6 (17.6%)	29 (40.8%)	
Type of resection				0.045 *
Partial gastrectomy	58 (55.2%)	14 (41.2%)	44 (62.0%)	
Total gastrectomy	47 (44.8%)	20 (58.8%)	27 (38.0%)	
Examined lymph nodes	34 (11–71)	35 (16–66)	34 (11–71)	0.813
Differentiation				0.496
Well differentiated	19 (18.1%)	4 (11.8%)	15 (21.1%)	
Moderately differentiated	36 (34.3%)	13 (38.2%)	23 (32.4%)	
Poorly differentiated	50 (47.6%)	17 (50.0%)	33 (46.5%)	
Postoperative complications				0.902
Yes	27 (25.7%)	9 (26.5%)	18 (25.4%)	
No	78 (74.3%)	25 (73.5%)	53 (74.6%)	
VAT SUVmean				<0.001 *
<0.41	52 (49.5%)	5 (14.7%)	47 (66.2%)	
≥0.41	53 (50.5%)	29 (85.3%)	24 (33.8%)	
SAT SUVmean				0.044 *
<0.33	52 (49.5%)	12 (35.3%)	40 (56.3%)	
≥0.33	53 (50.5%)	22 (64.7%)	31 (43.7%)	
GLIM criteria				<0.001 *
Malnutrition	41 (39.0%)	27 (79.4%)	14 (19.7%)	
No malnutrition	64 (61.0%)	7 (20.6%)	57 (80.3%)	
ALB (g/L)	43.1 (30.0–53.2)	41.9 (30.0–48.1)	43.8 (33.3–53.2)	<0.001 *
Pre-ALB (mg/L)	229 (64–422)	218 (64–383)	248 (144–422)	0.002 *
NLR	2.9 (1.1–13.0)	3.44 (1.7–13.0)	2.6 (1.1–12.3)	0.004 *
PLR	163.5 (61.0–443.8)	196.8 (103.6–419.1)	142.54 (61.0–443.8)	<0.001 *
CRP (mg/L)	2.0 (0.1–58.9)	2.73 (0.3–58.9)	1.78 (0.12–52.64)	0.009 *

Data are expressed as patient number (%) or median (range). * *p*-value < 0.05.

**Table 2:** Correlation analysis of VAT SUVmean and SAT SUVmean with clinical data.

	VAT SUVmean	SAT SUVmean
BMI		
r	−0.373	−0.165
*p*	<0.001	0.093
NLR		
r	0.306	0.06
*p*	0.001	0.541
PLR		
r	0.292	0.111
*p*	0.003	0.262
CRP		
r	0.061	0.114
*p*	0.536	0.246
ALB		
r	−0.331	−0.208
*p*	<0.001	0.034
Pre-ALB		
r	−0.360	−0.241
*p*	<0.001	0.013

**Table 3:** Univariate and multivariate Cox regression analyses for recurrence-free survival.

	Univariate Analysis		Multivariate Analysis	
Variables	Hazard Ratio (95% CI)	*p*-Value	Hazard Ratio (95% CI)	*p*-Value
Age	1.017 (0.990–1.046)	0.216		
BMI	0.862 (0.758–0.980)	0.023 *	1.070 (0.923–1.240)	0.369
Sex		0.507		
Women	1			
Men	0.773 (0.360–1.657)			
AJCC stage		<0.001 *		0.423
I-II	1		1	
III	4.446 (2.005–9.858)		1.588 (0.513–4.914)	
Adjuvant chemotherapy		0.018 *		0.170
No	1		1	
Yes	2.914 (1.205–7.049)		2.103 (0.727–6.087)	
Type of resection		0.031 *		0.482
Partial gastrectomy	1		1	
Total gastrectomy	2.123 (1.070–4.213)		2.103(0.727–6.087)	
Examined lymph nodes	1.013 (0.981–1.045)	0.436		
Differentiation		0.416		
Poorly differentiated	1			
Well differentiated Moderately differentiated	0.827 (0.523–1.308)			
Postoperative complications		0.633		
No	1			
Yes	1.205 (0.561–2.591)			
VAT SUVmean		<0.001 *		0.042 *
<0.41	1		1	
≥0.41	8.905 (3.404–23.300)		3.377(1.043–10.933)	
SAT SUVmean		0.034 *		0.434
<0.33	1		1	
≥0.33	2.142 (1.058–4.334)		1.383(0.614–3.115)	
GLIM criteria		<0.001 *		0.020 *
No malnutrition	1		1	
Malnutrition	9.259 (4.003–21.419)		4.731 (1.281–17.473)	
ALB	0.889 (0.828–0.954)	0.001 *	0.994 (0.895–1.104)	0.911
Pre-ALB	0.988 (0.982–0.995)	<0.001 *	0.998 (0.990–1.006)	0.656
NLR	1.163 (1.029–1.316)	0.016 *	1.125 (0.913–1.386)	0.269
PLR	1.004 (1.001–1.008)	0.006 *	1.000 (0.995–1.005)	0.988
CRP	1.017 (0.989–1.046)	0.229		

* *p*-value < 0.05.

**Table 4:** Comparisons of recurrence-free survival according to the combination of GLIM criteria and VAT SUVmean.

Patient Subgroup	Recurrence (%)	*p*-Value	Hazard Ratio (95% CI)
No malnutrition and VAT SUVmean < 0.41 (n = 42)	3 (8.8)	-	1.00
Malnutrition or VAT SUVmean ≥ 0.41 (n = 32)	6 (17.6)	<0.001	5.243 (2.581–10.65)
Malnutrition and VAT SUVmean ≥ 0.41 (n = 31)	25 (73.6%)	<0.001	18.41 (8.276–40.96)

**Table 5:** Comparison of the performance of different predictive models.

Model	LRT		AIC	C-Index
	χ^2^	*p*		
VAT SUVmean	29.67	<0.001	257.91	0.706
GLIM criteria	36.15	<0.001	251.43	0.766
VAT SUVmean and GLIM criteria	46.84	<0.001	242.74	0.802

## Data Availability

The datasets generated during and/or analyzed during the current study are available from the corresponding authors upon reasonable request.

## Abbreviations

The following abbreviations are used in this manuscript:

VAT	visceral adipose tissue
SAT	subcutaneous adipose tissue
SUV	standardized uptake value
GLIM	Global Leadership Initiative on Malnutrition
ALB	albumin
Pre-ALB	prealbumin
NLR	neutrophil-to-lymphocyte ratio
PLR	platelet-to-lymphocyte ratio
CRP	CRP
BMI	body mass index
CI	confidence interval
